# (2*E*)-2-(5-Bromo-2-hy­droxy-3-meth­oxy­benzyl­idene)-*N*-cyclo­hexyl­hydrazine­carbothio­amide

**DOI:** 10.1107/S1600536812007039

**Published:** 2012-02-24

**Authors:** Jinsa Mary Jacob, M. R. Prathapachandra Kurup

**Affiliations:** a Department of Applied Chemistry, Cochin University of Science and Technology, Kochi 682 022, India

## Abstract

The title compound, C_15_H_20_BrN_3_O_2_S, crystallizes in the thio­amide form and adopts an *E*,*E* conformation with respect to the azomethine and hydrazinic bonds, respectively. The mol­ecules are paired through N—H⋯O and O—H⋯S hydrogen bonds, leading to the formation of centrosymmetric dimers in the crystal. These dimers are stacked along the *a* axis and are inter­connected through N—H⋯S hydrogen bonds to generate polymeric chains. The structure also features C—H⋯π interactions. An intra­molecular O—H⋯O bond is also present.

## Related literature

For applications of hydrazinecarbothio­amide and its derivatives, see: Barber *et al.* (1992 ▶); Parrilha *et al.* (2011 ▶). For the synthesis, see: Klayman *et al.* (1979 ▶). For related structures, see: Dutta *et al.* (1997 ▶); Seena *et al.* (2006 ▶, 2008 ▶); Nisha *et al.* (2011 ▶). For standard bond-length data, see: Huheey *et al.* (1993 ▶); March (1992 ▶). For ring puckering analysis, see: Cremer & Pople (1975 ▶).
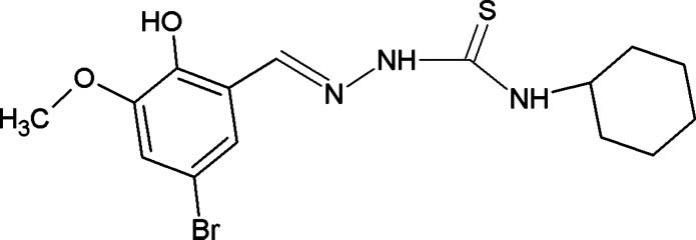


## Experimental

### 

#### Crystal data

C_15_H_20_BrN_3_O_2_S
*M* *_r_* = 386.31
Triclinic, 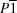

*a* = 5.7883 (4) Å
*b* = 11.412 (1) Å
*c* = 13.1312 (12) Å
α = 75.194 (4)°
β = 86.493 (3)°
γ = 83.489 (3)°
*V* = 832.72 (12) Å^3^
*Z* = 2
Mo *K*α radiation
μ = 2.60 mm^−1^
*T* = 296 K
0.30 × 0.25 × 0.25 mm

#### Data collection

Bruker APEXII CCD diffractometer
Absorption correction: multi-scan (*SADABS*; Bruker, 2004 ▶) *T*_min_ = 0.509, *T*_max_ = 0.562
12149 measured reflections
2923 independent reflections
2521 reflections with *I* > 2σ(*I*)
*R*_int_ = 0.070

#### Refinement

*R*[*F* ^2^ > 2σ(*F* ^2^)] = 0.035
*wR*(*F* ^2^) = 0.098
*S* = 1.12
2923 reflections
213 parameters
3 restraints
H atoms treated by a mixture of independent and constrained refinement
Δρ_max_ = 0.27 e Å^−3^
Δρ_min_ = −0.41 e Å^−3^

### 

Data collection: *APEX2* (Bruker, 2004 ▶); cell refinement: *APEX2* and *SAINT* (Bruker, 2004 ▶); data reduction: *SAINT* and *XPREP* (Bruker, 2004 ▶); program(s) used to solve structure: *SIR92* (Altomare *et al.*, 1993 ▶); program(s) used to refine structure: *SHELXL97* (Sheldrick, 2008 ▶); molecular graphics: *SHELXTL* (Sheldrick, 2008 ▶) and *ORTEP-3* (Farrugia, 1997 ▶); software used to prepare material for publication: *SHELXL97* and *publCIF* (Westrip, 2010 ▶).

## Supplementary Material

Crystal structure: contains datablock(s) global, I. DOI: 10.1107/S1600536812007039/fj2514sup1.cif

Structure factors: contains datablock(s) I. DOI: 10.1107/S1600536812007039/fj2514Isup2.hkl

Supplementary material file. DOI: 10.1107/S1600536812007039/fj2514Isup3.cml

Additional supplementary materials:  crystallographic information; 3D view; checkCIF report

## Figures and Tables

**Table 1:** Hydrogen-bond geometry (Å, °) *Cg*1 is the centroid of the C1–C6 ring.

*D*—H⋯*A*	*D*—H	H⋯*A*	*D*⋯*A*	*D*—H⋯*A*
O2—H2′⋯O1	0.83 (2)	2.21 (4)	2.616 (3)	110 (3)
O2—H2′⋯S1^i^	0.83 (2)	2.43 (3)	3.142 (2)	145 (3)
N2—H2⋯O2^i^	0.84 (2)	2.25 (2)	2.959 (3)	142 (3)
N3—H3′⋯S1^ii^	0.84 (2)	2.81 (3)	3.483 (3)	138 (3)
C13—H13*A*⋯*Cg*1^iii^	0.97	2.71	3.664 (4)	168

Symmetry codes: (i) 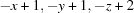; (ii) 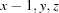; (iii) 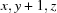.

**Fig. 1.**
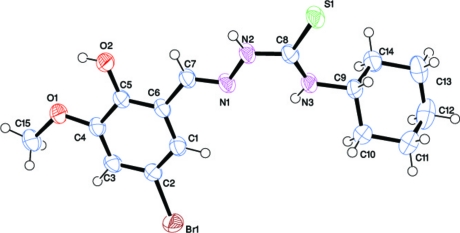
The title compound, showing 50% probability displacement ellipsoids and the atom-numbering scheme.

**Fig. 2.**
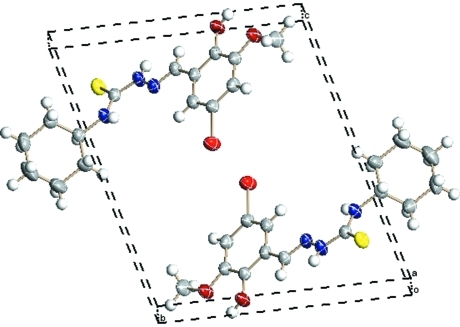
A view of the unit cell along a axis.

## supplementary crystallographic information

### Comment

The hydrazinecarbothioamides of aromatic aldehydes and ketones have been shown to possess a diverse range of biological activities (Parrilha *et al.*, 2011) and catalytic activity (Barber *et al.*, 1992). The pharmacological activity of hydrazinecarbothioamides of *o*-hydroxyaromatic aldehydes is correlated to their ability to form chelates with biologically important metal ions by bonding through O, N and S atoms (Dutta *et al.*, 1997) and reductive capacity.

The title compound adopts an *E* configuration with respect to the C8—N2 bond (Fig. 1) similar to salicylaldehyde-*N*(4)-phenylthiosemicarbazone (Seena *et al.*, 2008) but in contrast to 2-hydroxyacetophenone-*N*(4)-phenylthiosemicarbazone (Seena *et al.*, 2006), where a *Z* configuration exists. This is confirmed by the N1—N2—C8—S1 torsion angle of -175.9 (2)°. Also *E* configuration is perceived about the azomethine bond [N2—N1—C7—C6 = -175.5 (3)°] (Nisha *et al.*, 2011). Atom O1 lies *cis* to O2, with an O1—C4—C5—O2 torsion angle of 0.7 (4)°. This favours the intramolecular hydrogen bonding interaction between O1 and H attached to O2 atom.

The C8—S1 bond distance [1.685 (3) Å] is closer to C═S bond length [1.60 Å] than to C—S bond length [1.81 Å] (Huheey *et al.*, 1993) which confirms the existence of the compound in the thioamido form in solid state. Also the C7—N1 bond distance [1.267 (4) Å] is appreciably close to that of a C═N double bond [1.28 Å] (March, 1992), confirming the azomethine bond formation.

The mean plane deviation calculations show that the molecule as a whole is non-planar. But the central hydrazinecarbothioamide group (C7/N1/N2/C8/S1/N3/C9) is almost planar with a maximum deviation from the mean plane of -0.054 (2) Å for atom N1. This is similar to that observed in salicylaldehyde-*N*(4)-phenyl thiosemicarbazone (Seena *et al.*, 2008). The ring *Cg*1^iii^ (comprising atoms C1—C6, with a maximum deviation of 0.005 (3) Å for C3) makes a dihedral angle of 18.90 (12)° with the hydrazinecarbothioamide moiety. Ring puckering analysis (Cremer & Pople, 1975) and least square plane calculations show that the cyclohexyl ring adopts a chair conformation (*Q_T_* = 0.568 (4) Å) with the equatorial substitution at C9 for N3.

Fig. 2 shows the packing diagram of the title compound. The crystal packing involves one intramolecular and three intermolecular hydrogen bonds (Table 1). The intramolecular hydrogen bonding interaction, O2—H2'···O1 leads to the formation of a five membered ring comprising of atoms C4, C5, O2, H2' and O1 and facilitates almost planar geometry in part of the molecule. The intermolecular hydrogen bonds N2—H2···O2^i^ and O2—H2'···S1^i^ cause the pairing of molecules leading to the formation of centrosymmetric dimers in the crystal lattice. These dimers are stacked along the *a* axis and are interconnected through a third intermolecular hydrogen bond N3—H3'···S1^ii^ to produce independent polymeric chains in the packing. Further stabilization is provided by C13—H13A···*Cg*1^iii^ interaction.

### Experimental

The preparation of this compound involves a two step process (Klayman *et al.*, 1979). In the first step, cyclohexyl isothiocyanate (15 mmol, 2 ml) in 15 ml methanol and hydrazine hydrate (90 mmol, 4.3 ml) in 15 ml methanol were mixed and the resulting solution was stirred for an hour. The white product, *N*(4)-cyclohexylthiosemicarbazide formed was filtered, washed with methanol and dried *in vacuo*. In the second step, a methanolic (20 ml) solution of 4-cyclohexylthiosemicarbazide (1 mmol, 0.1732 g) was added to a solution of 5-bromo-3-methoxysalicylaldehyde (1 mmol, 0.2310 g) in 15 ml methanol and the reaction mixture was refluxed for 2 h in acid medium. The product formed was filtered, washed with methanol and dried *in vacuo*. Suitable crystals were grown by slow evaporation of its solution in 1:1 mixture of DMF and methanol over 2 days.

### Refinement

All H atoms on C were placed in calculated positions, guided by difference maps, with C—H bond distances 0.93–0.97 Å. H atoms were assigned as *U*_iso_=1.2Ueq (1.5 for Me). N2—H2, N3—H3' and O2—H2' H atoms were located from difference maps and restrained using *DFIX* instructions.

### Crystal data

**Table:**

C_15_H_20_BrN_3_O_2_S	*Z* = 2
*M**_r_* = 386.31	*F*(000) = 396
Triclinic, *P*1	*D*_x_ = 1.541 Mg m^−^^3^
Hall symbol: -P 1	Mo *K*α radiation, λ = 0.71073 Å
*a* = 5.7883 (4) Å	Cell parameters from 6391 reflections
*b* = 11.412 (1) Å	θ = 2.7–27.9°
*c* = 13.1312 (12) Å	µ = 2.60 mm^−^^1^
α = 75.194 (4)°	*T* = 296 K
β = 86.493 (3)°	Block, colourless
γ = 83.489 (3)°	0.30 × 0.25 × 0.25 mm
*V* = 832.72 (12) Å^3^	

### Data collection

**Table:**

Bruker APEXII CCD diffractometer	2923 independent reflections
Radiation source: fine-focus sealed tube	2521 reflections with *I* > 2σ(*I*)
Graphite monochromator	*R*_int_ = 0.070
Detector resolution: 8.33 pixels mm^-1^	θ_max_ = 25.0°, θ_min_ = 2.7°
ω and φ scan	*h* = −6→6
Absorption correction: multi-scan (*SADABS*; Bruker, 2004)	*k* = −13→13
*T*_min_ = 0.509, *T*_max_ = 0.562	*l* = −15→15
12149 measured reflections	

### Refinement

**Table:**

Refinement on *F*^2^	Secondary atom site location: difference Fourier map
Least-squares matrix: full	Hydrogen site location: inferred from neighbouring sites
*R*[*F*^2^ > 2σ(*F*^2^)] = 0.035	H atoms treated by a mixture of independent and constrained refinement
*wR*(*F*^2^) = 0.098	*w* = 1/[σ^2^(*F*_o_^2^) + (0.0194*P*)^2^ + 0.6265*P*] where *P* = (*F*_o_^2^ + 2*F*_c_^2^)/3
*S* = 1.12	(Δ/σ)_max_ = 0.001
2923 reflections	Δρ_max_ = 0.27 e Å^−^^3^
213 parameters	Δρ_min_ = −0.41 e Å^−^^3^
3 restraints	Extinction correction: *SHELXL97* (Sheldrick, 2008), Fc^*^=kFc[1+0.001xFc^2^λ^3^/sin(2θ)]^-1/4^
Primary atom site location: structure-invariant direct methods	Extinction coefficient: 0.0142 (18)

### Special details

**Table:**

Geometry. All e.s.d.'s (except the e.s.d. in the dihedral angle between two l.s. planes) are estimated using the full covariance matrix. The cell e.s.d.'s are taken into account individually in the estimation of e.s.d.'s in distances, angles and torsion angles; correlations between e.s.d.'s in cell parameters are only used when they are defined by crystal symmetry. An approximate (isotropic) treatment of cell e.s.d.'s is used for estimating e.s.d.'s involving l.s. planes.
Refinement. Refinement of *F*^2^ against ALL reflections. The weighted *R*-factor *wR* and goodness of fit *S* are based on *F*^2^, conventional *R*-factors *R* are based on *F*, with *F* set to zero for negative *F*^2^. The threshold expression of *F*^2^ > σ(*F*^2^) is used only for calculating *R*-factors(gt) *etc*. and is not relevant to the choice of reflections for refinement. *R*-factors based on *F*^2^ are statistically about twice as large as those based on *F*, and *R*- factors based on ALL data will be even larger.

### Fractional atomic coordinates and isotropic or equivalent isotropic displacement parameters (Å^2^)

**Table:**

	*x*	*y*	*z*	*U*_iso_*/*U*_eq_	
Br1	−0.42662 (6)	0.51878 (3)	0.63154 (3)	0.05382 (17)	
S1	0.75115 (13)	0.88705 (7)	0.80347 (7)	0.0448 (2)	
O1	−0.1322 (4)	0.2029 (2)	0.97526 (18)	0.0520 (6)	
O2	0.1901 (4)	0.3362 (2)	0.99890 (19)	0.0471 (6)	
N1	0.2789 (4)	0.6672 (2)	0.8130 (2)	0.0377 (6)	
N2	0.4703 (4)	0.7170 (2)	0.8316 (2)	0.0398 (6)	
N3	0.3358 (5)	0.8967 (2)	0.7247 (2)	0.0407 (6)	
C1	−0.0696 (5)	0.5340 (3)	0.7638 (2)	0.0384 (7)	
H1	−0.0523	0.6089	0.7167	0.046*	
C2	−0.2371 (5)	0.4647 (3)	0.7502 (2)	0.0391 (7)	
C3	−0.2709 (5)	0.3530 (3)	0.8186 (2)	0.0405 (7)	
H3	−0.3877	0.3081	0.8082	0.049*	
C4	−0.1268 (5)	0.3105 (3)	0.9024 (2)	0.0379 (7)	
C5	0.0459 (5)	0.3795 (3)	0.9177 (2)	0.0358 (7)	
C6	0.0749 (5)	0.4910 (3)	0.8492 (2)	0.0360 (7)	
C7	0.2600 (5)	0.5588 (3)	0.8659 (2)	0.0370 (7)	
H7	0.3681	0.5218	0.9169	0.044*	
C8	0.5028 (5)	0.8331 (3)	0.7839 (2)	0.0350 (7)	
C9	0.3347 (5)	1.0238 (3)	0.6669 (2)	0.0387 (7)	
H9	0.4949	1.0399	0.6439	0.046*	
C10	0.1921 (7)	1.0438 (3)	0.5704 (3)	0.0517 (9)	
H10A	0.0369	1.0206	0.5918	0.062*	
H10B	0.2626	0.9922	0.5262	0.062*	
C11	0.1758 (8)	1.1755 (4)	0.5079 (3)	0.0687 (11)	
H11A	0.3286	1.1957	0.4788	0.082*	
H11B	0.0734	1.1866	0.4496	0.082*	
C12	0.0842 (8)	1.2596 (4)	0.5760 (4)	0.0710 (12)	
H12A	−0.0747	1.2451	0.5992	0.085*	
H12B	0.0838	1.3435	0.5351	0.085*	
C13	0.2320 (7)	1.2397 (3)	0.6707 (3)	0.0602 (10)	
H13A	0.1675	1.2930	0.7145	0.072*	
H13B	0.3882	1.2601	0.6476	0.072*	
C14	0.2423 (6)	1.1085 (3)	0.7344 (3)	0.0481 (8)	
H14A	0.3423	1.0969	0.7936	0.058*	
H14B	0.0878	1.0898	0.7620	0.058*	
C15	−0.2900 (7)	0.1217 (3)	0.9629 (3)	0.0551 (9)	
H15A	−0.4466	0.1577	0.9693	0.083*	
H15B	−0.2659	0.0468	1.0165	0.083*	
H15C	−0.2643	0.1055	0.8947	0.083*	
H3'	0.211 (4)	0.866 (3)	0.723 (3)	0.041 (9)*	
H2	0.576 (5)	0.672 (3)	0.868 (2)	0.040 (9)*	
H2'	0.144 (6)	0.277 (2)	1.042 (2)	0.055 (11)*	

### Atomic displacement parameters (Å^2^)

**Table:**

	*U*^11^	*U*^22^	*U*^33^	*U*^12^	*U*^13^	*U*^23^
Br1	0.0582 (3)	0.0553 (3)	0.0480 (2)	−0.01859 (17)	−0.01714 (16)	−0.00386 (17)
S1	0.0339 (4)	0.0390 (5)	0.0610 (5)	−0.0161 (3)	−0.0082 (4)	−0.0048 (4)
O1	0.0655 (15)	0.0405 (13)	0.0502 (14)	−0.0291 (11)	−0.0122 (11)	0.0010 (11)
O2	0.0522 (14)	0.0394 (13)	0.0484 (14)	−0.0181 (11)	−0.0168 (11)	0.0008 (11)
N1	0.0335 (13)	0.0323 (14)	0.0491 (15)	−0.0107 (10)	−0.0055 (11)	−0.0096 (12)
N2	0.0335 (14)	0.0287 (14)	0.0568 (17)	−0.0083 (11)	−0.0134 (12)	−0.0050 (12)
N3	0.0372 (15)	0.0336 (14)	0.0506 (16)	−0.0158 (11)	−0.0100 (12)	−0.0019 (12)
C1	0.0387 (16)	0.0334 (16)	0.0433 (17)	−0.0078 (13)	−0.0013 (13)	−0.0081 (14)
C2	0.0411 (17)	0.0388 (17)	0.0390 (17)	−0.0107 (13)	−0.0054 (13)	−0.0088 (14)
C3	0.0400 (17)	0.0419 (18)	0.0435 (18)	−0.0168 (13)	−0.0023 (13)	−0.0123 (14)
C4	0.0413 (17)	0.0338 (16)	0.0396 (17)	−0.0147 (13)	0.0013 (13)	−0.0068 (13)
C5	0.0368 (16)	0.0323 (16)	0.0402 (17)	−0.0090 (12)	−0.0027 (13)	−0.0097 (13)
C6	0.0351 (16)	0.0311 (16)	0.0443 (17)	−0.0081 (12)	−0.0020 (13)	−0.0117 (13)
C7	0.0358 (16)	0.0312 (16)	0.0451 (18)	−0.0089 (12)	−0.0047 (13)	−0.0083 (14)
C8	0.0333 (15)	0.0342 (16)	0.0393 (17)	−0.0099 (12)	0.0014 (12)	−0.0103 (13)
C9	0.0399 (16)	0.0325 (16)	0.0429 (17)	−0.0129 (12)	−0.0049 (13)	−0.0030 (13)
C10	0.066 (2)	0.044 (2)	0.0432 (19)	−0.0136 (16)	−0.0122 (16)	−0.0028 (15)
C11	0.094 (3)	0.050 (2)	0.054 (2)	−0.014 (2)	−0.016 (2)	0.0069 (19)
C12	0.067 (3)	0.044 (2)	0.086 (3)	−0.0003 (18)	0.001 (2)	0.010 (2)
C13	0.071 (3)	0.0349 (19)	0.075 (3)	−0.0115 (17)	0.014 (2)	−0.0166 (18)
C14	0.057 (2)	0.0419 (19)	0.048 (2)	−0.0148 (15)	0.0006 (16)	−0.0106 (15)
C15	0.064 (2)	0.044 (2)	0.060 (2)	−0.0312 (17)	−0.0025 (18)	−0.0071 (17)

### Geometric parameters (Å, º)

**Table:**

Br1—C2	1.891 (3)	C7—H7	0.9300
S1—C8	1.685 (3)	C9—C14	1.506 (4)
O1—C4	1.352 (4)	C9—C10	1.508 (4)
O1—C15	1.417 (4)	C9—H9	0.9800
O2—C5	1.351 (4)	C10—C11	1.513 (5)
O2—H2'	0.826 (19)	C10—H10A	0.9700
N1—C7	1.267 (4)	C10—H10B	0.9700
N1—N2	1.363 (3)	C11—C12	1.507 (6)
N2—C8	1.342 (4)	C11—H11A	0.9700
N2—H2	0.840 (18)	C11—H11B	0.9700
N3—C8	1.308 (4)	C12—C13	1.507 (6)
N3—C9	1.454 (4)	C12—H12A	0.9700
N3—H3'	0.842 (18)	C12—H12B	0.9700
C1—C2	1.366 (4)	C13—C14	1.513 (5)
C1—C6	1.392 (4)	C13—H13A	0.9700
C1—H1	0.9300	C13—H13B	0.9700
C2—C3	1.385 (4)	C14—H14A	0.9700
C3—C4	1.374 (4)	C14—H14B	0.9700
C3—H3	0.9300	C15—H15A	0.9600
C4—C5	1.394 (4)	C15—H15B	0.9600
C5—C6	1.379 (4)	C15—H15C	0.9600
C6—C7	1.449 (4)		
			
C4—O1—C15	118.5 (3)	C10—C9—H9	108.5
C5—O2—H2'	113 (3)	C9—C10—C11	111.4 (3)
C7—N1—N2	115.6 (3)	C9—C10—H10A	109.3
C8—N2—N1	120.9 (2)	C11—C10—H10A	109.3
C8—N2—H2	120 (2)	C9—C10—H10B	109.3
N1—N2—H2	119 (2)	C11—C10—H10B	109.3
C8—N3—C9	125.6 (2)	H10A—C10—H10B	108.0
C8—N3—H3'	119 (2)	C12—C11—C10	111.1 (3)
C9—N3—H3'	115 (2)	C12—C11—H11A	109.4
C2—C1—C6	119.1 (3)	C10—C11—H11A	109.4
C2—C1—H1	120.4	C12—C11—H11B	109.4
C6—C1—H1	120.4	C10—C11—H11B	109.4
C1—C2—C3	122.6 (3)	H11A—C11—H11B	108.0
C1—C2—Br1	119.6 (2)	C11—C12—C13	110.8 (3)
C3—C2—Br1	117.8 (2)	C11—C12—H12A	109.5
C4—C3—C2	118.1 (3)	C13—C12—H12A	109.5
C4—C3—H3	120.9	C11—C12—H12B	109.5
C2—C3—H3	120.9	C13—C12—H12B	109.5
O1—C4—C3	126.0 (3)	H12A—C12—H12B	108.1
O1—C4—C5	113.8 (3)	C12—C13—C14	110.8 (3)
C3—C4—C5	120.2 (3)	C12—C13—H13A	109.5
O2—C5—C6	119.3 (3)	C14—C13—H13A	109.5
O2—C5—C4	120.0 (3)	C12—C13—H13B	109.5
C6—C5—C4	120.7 (3)	C14—C13—H13B	109.5
C5—C6—C1	119.2 (3)	H13A—C13—H13B	108.1
C5—C6—C7	119.2 (3)	C9—C14—C13	110.4 (3)
C1—C6—C7	121.5 (3)	C9—C14—H14A	109.6
N1—C7—C6	121.9 (3)	C13—C14—H14A	109.6
N1—C7—H7	119.1	C9—C14—H14B	109.6
C6—C7—H7	119.1	C13—C14—H14B	109.6
N3—C8—N2	116.6 (3)	H14A—C14—H14B	108.1
N3—C8—S1	124.5 (2)	O1—C15—H15A	109.5
N2—C8—S1	118.8 (2)	O1—C15—H15B	109.5
N3—C9—C14	111.7 (3)	H15A—C15—H15B	109.5
N3—C9—C10	108.2 (2)	O1—C15—H15C	109.5
C14—C9—C10	111.4 (3)	H15A—C15—H15C	109.5
N3—C9—H9	108.5	H15B—C15—H15C	109.5
C14—C9—H9	108.5		
			
C7—N1—N2—C8	−176.8 (3)	C2—C1—C6—C7	178.1 (3)
C6—C1—C2—C3	0.5 (5)	N2—N1—C7—C6	−175.5 (3)
C6—C1—C2—Br1	−177.5 (2)	C5—C6—C7—N1	−172.0 (3)
C1—C2—C3—C4	−1.0 (5)	C1—C6—C7—N1	10.1 (5)
Br1—C2—C3—C4	177.1 (2)	C9—N3—C8—N2	179.5 (3)
C15—O1—C4—C3	3.8 (5)	C9—N3—C8—S1	−0.6 (5)
C15—O1—C4—C5	−175.5 (3)	N1—N2—C8—N3	4.1 (4)
C2—C3—C4—O1	−178.6 (3)	N1—N2—C8—S1	−175.9 (2)
C2—C3—C4—C5	0.7 (5)	C8—N3—C9—C14	−86.2 (4)
O1—C4—C5—O2	0.7 (4)	C8—N3—C9—C10	150.8 (3)
C3—C4—C5—O2	−178.7 (3)	N3—C9—C10—C11	178.1 (3)
O1—C4—C5—C6	179.4 (3)	C14—C9—C10—C11	54.9 (4)
C3—C4—C5—C6	0.0 (5)	C9—C10—C11—C12	−54.6 (5)
O2—C5—C6—C1	178.2 (3)	C10—C11—C12—C13	55.9 (5)
C4—C5—C6—C1	−0.4 (5)	C11—C12—C13—C14	−57.4 (4)
O2—C5—C6—C7	0.2 (4)	N3—C9—C14—C13	−177.2 (3)
C4—C5—C6—C7	−178.4 (3)	C10—C9—C14—C13	−56.0 (4)
C2—C1—C6—C5	0.2 (5)	C12—C13—C14—C9	57.3 (4)

### Hydrogen-bond geometry (Å, º)

*Cg*1 is the centroid of the C1-C6 ring.

**Table:**

*D*—H···*A*	*D*—H	H···*A*	*D*···*A*	*D*—H···*A*
O2—H2′···O1	0.83 (2)	2.21 (4)	2.616 (3)	110 (3)
O2—H2′···S1^i^	0.83 (2)	2.43 (3)	3.142 (2)	145 (3)
N2—H2···O2^i^	0.84 (2)	2.25 (2)	2.959 (3)	142 (3)
N3—H3′···S1^ii^	0.84 (2)	2.81 (3)	3.483 (3)	138 (3)
C13—H13*A*···*Cg*1^iii^	0.97	2.71	3.664 (4)	168

Symmetry codes: (i) −*x*+1, −*y*+1, −*z*+2; (ii) *x*−1, *y*, *z*; (iii) *x*, *y*+1, *z*.
